# A new biostimulant derived from soybean by-products enhances plant tolerance to abiotic stress triggered by ozone

**DOI:** 10.1186/s12870-024-05290-3

**Published:** 2024-06-19

**Authors:** Angel Orts, Salvadora Navarro-Torre, Sandra Macías-Benítez, José M. Orts, Emilia Naranjo, Angélica Castaño, Juan Parrado

**Affiliations:** 1 Departamento de Bioquímica y Biología Molecular, Facultad de Farmacia, Universidad de Sevilla. C/Profesor García González, Nº2. 41012 Seville, Spain; 2 Departamento de Microbiología y Parasitología, Facultad de Farmacia, Universidad de Sevilla. C/Profesor García González, Nº2. 41012 Seville, Spain

**Keywords:** Biostimulant, Capsicum, Enzymatic extract, Okara, Ozone, ROS

## Abstract

**Background:**

Tropospheric ozone is an air pollutant that causes negative effects on vegetation, leading to significant losses in crop productivity. It is generated by chemical reactions in the presence of sunlight between primary pollutants resulting from human activity, such as nitrogen oxides and volatile organic compounds. Due to the constantly increasing emission of ozone precursors, together with the influence of a warming climate on ozone levels, crop losses may be aggravated in the future. Therefore, the search for solutions to mitigate these losses becomes a priority.

Ozone-induced abiotic stress is mainly due to reactive oxygen species generated by the spontaneous decomposition of ozone once it reaches the apoplast. In this regard, compounds with antioxidant activity offer a viable option to alleviate ozone-induced damage. Using enzymatic technology, we have developed a process that enables the production of an extract with biostimulant properties from okara, an industrial soybean byproduct. The biostimulant, named as OEE (Okara Enzymatic Extract), is water-soluble and is enriched in bioactive compounds present in okara, such as isoflavones. Additionally, it contains a significant fraction of protein hydrolysates contributing to its functional effect.

Given its antioxidant capacity, we aimed to investigate whether OEE could alleviate ozone-induced damage in plants. For that, pepper plants (*Capsicum annuum*) exposed to ozone were treated with a foliar application of OEE.

**Results:**

OEE mitigated ozone-induced damage, as evidenced by the net photosynthetic rate, electron transport rate, effective quantum yield of PSII, and delayed fluorescence. This protection was confirmed by the level of expression of genes associated with photosystem II. The beneficial effect was primarily due to its antioxidant activity, as evidenced by the lipid peroxidation rate measured through malondialdehyde content. Additionally, OEE triggered a mild oxidative response, indicated by increased activities of antioxidant enzymes in leaves (catalase, superoxide dismutase, and guaiacol peroxidase) and the oxidative stress index, providing further protection against ozone-induced stress.

**Conclusions:**

The present results support that OEE protects plants from ozone exposure. Taking into consideration that the promotion of plant resistance against abiotic damage is an important goal of biostimulants, we assume that its use as a new biostimulant could be considered.

## Introduction

Tropospheric ozone (O_3_) is a major air pollutant that induces abiotic stress in plants causing negative effects on growth and crop productivity [1–3]. Differently from other air pollutants, tropospheric ozone is not emitted directly but is generated from primary pollutants resulting from human activity, such as methane, carbon monoxide, nitrogen oxides and volatile organic compounds, through chemical reactions in the presence of sunlight [4]. As a result, the tropospheric ozone levels are constantly increasing due to increased human activity. Despite the authorities' interest in controlling pollutant emissions, global warming is likely to contribute to increasing concentrations of tropospheric ozone over time, leading to a greater loss of agricultural productivity [5–7].

The damage induced by ozone in living organisms is due to its high oxidizing power. The adverse impacts of O_3_ on plants encompass a reduction in photosynthesis, increased water loss, and the development of chlorotic and necrotic spots on leaves [2, 8]. Ozone-induced damage primarily arises from the generation of reactive oxygen species (ROS) through the spontaneous decomposition of O_3_ upon entering the apoplastic space or through direct interactions with various cellular components. This process triggers oxidative damage to biomolecules, potentially affecting crucial cellular functions [9].

The loss in crop productivity and its impact on global food supply [3, 10–12] have heightened the interest in finding strategic solutions to minimize this loss in areas with elevated concentrations of tropospheric O_3_. While one possible solution is the use of genetically modified organisms, social rejection of their use drives the search for alternative solutions [13]. An alternative approach involves the application of exogenous compounds that protect plants against O_3_.

Compounds of various natures, including antioxidants, herbicides, pesticides, plant growth regulators or mechanical barriers have been used to protect plants from ozone damage, either alone or in combination. However, despite achieving a certain degree of protection in some cases, the side effects render their use in the field unacceptable [14]. To date, the antioxidant EDU (ethylene diurea– (N-[2-(2-oxo-1-imidazolidinyl) ethyl]-N0 phenylurea), has been considered the most effective antiozonant. However, its primary mechanism of action remains unclear, and the use of EDU is advised primarily as a research tool for evaluating the phytotoxic effects of O_3_ on crops but it has not yet been implemented at the commercial level [14].

Therefore, it is essential to seek new alternatives that allow overcoming these inconveniences. In this context, bio-stimulants derived from plant extracts emerge as a good option since they do not generate environmental toxicity. According to the European Biostimulant Industry Council (EBIC), biostimulants are defined as "substances and/or microorganisms that, when applied to plants or the rhizosphere, stimulate natural processes, enhancing nutrient absorption, nutritional efficiency, abiotic stress tolerance, and crop quality.

Through the use of enzymatic technology, our group has already developed a rice bran extract with bio-stimulant properties that protected pepper plants against acute exposure to O_3_ [15]. Thus, we have extended this technology to other agricultural by-products such as the soy pulp, also called okara.

Okara is a byproduct generated during the production of soy milk and tofu. Despite having a rich composition of nutrients, such as proteins of high-quality, fiber, fats, and carbohydrates, as well as containing bioactive compounds like isoflavones, two inconveniences contribute to its underutilization. First, the susceptibility to rapid deterioration due to high moisture content, and second its low water solubility [16, 17]. Through the application of enzymatic technology, our team has developed a process that facilitates the creation of a stable, water-soluble enzymatic extract from okara, OEE. This extract retains most of the isoflavones from the original okara while being rich in bioactive peptides, all of which contribute to its high antioxidant potency [18].

The elevated level of bioactive compounds present in OEE and its potent antioxidant activity has prompted us to explore its potential as plant biostimulant. Similar to our previous work [15], we opted for pepper plants (*Capsicum annuum*) for this research due to the global significance of the pepper crop. Capsicum pepper is among the most widely cultivated vegetable crops internationally, experiencing a substantial increase in total production in recent years, reaching around 40 million tons in 2020 [19]. These plants are predominantly grown in subtropical regions globally, with major producers situated in developing countries like China, India, or Mexico, where O_3_ concentrations are anticipated to rise more significantly than in other nations. Due to these factors, capsicum peppers serve as a compelling subject for studying the adverse effects of O_3_.

To evaluate OEE biostimulant capacity, pepper plants exposed to O_3_ were treated with a foliar spray of an aqueous solution of OEE. The protective effect was analyzed through the assessment of biochemical parameters such as antioxidant enzymes in leaves (catalase, superoxide dismutase, and guaiacol peroxidase) or lipid peroxidation rate (MDA) as well as key plants physiological parameters such as net photosynthetic rate (A_N_), electron transport rate (ETR), effective quantum yield of PSII (PhiPSII), and delayed fluorescence (DF). Additionally, genes related to photosystem II was also evaluated.

## Material and methods

### Okara extract preparation

Okara, obtained from Soria Natural S.L. (Garray, Soria, Spain), was prepared by washing soybeans, soaking them in cold water (33.3% w/v) for 30 h, and then heating them at 95 °C for 5 min to deactivate trypsin and lipoxygenase inhibitors. The soaked soybeans were ground with hot water in a 1:1 (w/v) ratio to produce soy milk through pressure and filtration, leaving behind soy pulp known as okara.

The enzymatic hydrolysis of okara was carried out using a liquid enzyme serine-endoprotease subtilisin (EC 3.4.21.62) from enterprise Biocon (Spain). The preparation of okara was a 10% concentration in water (dry w/v), and protein hydrolysis was conducted at pH 10.0 using the pH–stat method. The process included sequential incubation with subtilisin (0.3% v/v) for 2 h at 55 °C without shaking. After centrifugation for 40 min at 4 °C and 10,000 g, the soluble phase (OEE) was heat-dried and analyzed, while the sediment was weighed and discarded.

The nutritional composition of hydrolyzed okara was characterized for macro- and micronutrients as described in previous work by our group [18].

The protein content of the soluble portion of okara was analyzed by size-exclusion chromatography on an ÄKTA-purifier FPLC system (GE Healthcare), filtration chromatography and a Superdex Peptide 10/300GL column. This column has an exclusion range of 700 to 10,000 Da, which separates free peptides and amino acids. After centrifugation, the supernatant underwent filtration through a 0.2 μm filter and was then loaded into a 0.1 mL loop connected to an ÄKTA-purifier system. The column was equilibrated and eluted with 0.25 M Tris–HCl buffer (pH 7.0) using isocratic mode at a flow rate of 0.5 mL/min. Protein and peptide detection occurred at 280 and 215 nm, respectively, using a GE Healthcare UV900 module coupled to the column elution.

### Plant Treatment

The selected plants and the treatment applied were carried out according to previous work by the group [15]*. Capsicum annum* L. var. *grossum* (pepper) plants were raised from seeds in plastic pots containing an organic commercial substrate (Gramoflor GmbH und Co. KG.) and Osmocote® (NPK 15: 9: 12), and grown inside the University of Seville Glasshouse General Services on a phytoclimatic chamber, Fitoclima 18,000 PHL (Aralab-Spain), with a controlled temperature of 18 − 22 °C, 50% relative humidity, adequate irrigation with tap water and a photoperiod of 16 h light/8 h darkness, being the maximum photosynthetic photon flux density level incident on leaves of 1200 μmol m^−2^ s^−1^.

After eight days of transplantation, 20 pepper plants were selected and divided in 4 groups (5 plants for group): control plants (Ct), control plants under O_3_ exposition (Ct + O_3_), plants treated with OEE (OEE) and plants treated with OEE under O_3_ exposition (OEE + O_3_). Following protocol previously describe by us [15], to evaluate the protection capacity of the treatment with OEE, plants were foliar sprayed a total of 4 times at five-day intervals, with an aqueous solution of OEE at 0.1% (groups OEE and OEE + O_3_) or distilled water (groups Ct and Ct + O_3_). After 5 days of the last spray treatment, Ct + O_3_ and OEE + O_3_ plants were transferred to another phytoclimatic chamber with an ozone generator (ZONOSISTEM GM 5000 O_3_ Generator) attached and exposed to 3 consecutive fumigations with 100 ppb of O_3_ for 6 h (from 10:00 am to 4:00 pm). After ozone fumigation all the test plants were sprayed again with the corresponding solution (OEE 0.1% or distilled water).

Finally, 24 h after the last exposure to ozone, foliar samples were taken from each plant and the analyses described below were carried out.

### Plants status after the ozone exposition

#### Analyses of photosynthetic parameters

Twenty-four hours after the last ozone treatment, net photosynthetic rate (A_N_), electron transport rate (ETR) and effective quantum yield of PSII (PhiPS2) were determined in plants using an IRGA (LI-6400XT, LI-COR Inc., Nev., EEUU) with a light chamber for the leaf (Li-6400-02B, Li-Cor Inc.) according to Macias-Benitez et al. [15]. Briefly, measurements (*n* = 20) were performed between 10 a.m. and 2 p.m. hours under a photosynthetic photon flux density of 1500 \upmu mol.m^−2^. s^−1^, a deficit of vapor pressure of 2–3 kPa, a temperature around 25ºC, and a CO_2_ concentration environment of 400 \upmu mol.mol^−1^ air. Each measurement was recorded after the stabilization of the exchange of gases was equilibrated (120 s).

#### Delayed fluorescence determination

Delayed fluorescence (DF) was recorded at the end of the experiment in a random leaf from each plant. For that, the collected leaves were analyzed using a NightShade LB 985 (Berthold Technologies, Germany) equipped with a deeply cooled CCD camera [20]. The recorded data were converted to counts per second (cps) and normalized to the leaf area.

### RNAseq

#### Sample Collection

The sample collection was carried out following the protocol provided by the company Corning.

#### Extraction, purification of Samples and library Preparation

The extraction and purification of the input RNA was performed by GENEWIZ Multiomics & Synthesis Solutions from Azenta Life Sciences.

#### Mapping sequence reads to the reference genome

Sequence reads were trimmed to remove possible adapter sequences and nucleotides with poor quality using Trimmomatic v.0.36. The trimmed reads were mapped to the capsicum_annuum reference genome available on ENSEMBL using the STAR aligner v.2.5.2b. The STAR aligner is a splice aligner that detects splice junctions and incorporates them to help align the entire read sequences. BAM files were generated because of this step.

#### Extracting gene hit counts

Unique gene hit counts were calculated by using feature counts from the Subread package v.1.5.2. The hit counts were summarized and reported using the gene_id feature in the annotation file. Only unique reads that fell within exon regions were counted.

#### Differential gene expression analysis

After extraction of gene hit counts, the gene hit counts table was used for downstream differential expression analysis. Using DESeq2, a comparison of gene expression between the customer-defined groups of samples was performed. The Wald test was used to generate *p*-values and log2 fold changes. Genes with an adjusted *p*-value < 0.05 and absolute log2 fold change > 1 were called as differentially expressed genes for each comparison.

#### Bioinformatics tools for functional analysis

To verify the annotation, and thus the function of the overexpressed genes and proteins, the gene ontology provided by UniprotKB, annotations from NCBI, PATRIC, and Ecogene were consulted, as well as the gene ontology assigned by the JCVI Microbial Resource Center. Additionally, these genes and proteins were sorted according to the orthologous classification provided by KEGG [21], incorporating into this classification those genes and proteins reviewed by the various annotations and ontologies mentioned earlier. As the first functional analysis, the different functional categories described in the clusters of orthologous groups (COG) associated with each overexpressed gene or protein were consulted.

### Oxidative stress level in plants after the ozone exposition

#### Antioxidant enzyme analysis

In the same way of the determination of lipid peroxidation, a pool of leaves was created and collected in liquid nitrogen and stored at -80˚C until the analysis.

Extraction was carried out using a 50 mM sodium phosphate buffer (pH 7.6). After samples homogenization and centrifugation at 4˚C, the total protein content in extracts was determined according to Bradford protocol [22].

The analysis of the antioxidant enzymes (catalase (CAT), superoxide dismutase (SOD), and guaiacol peroxidase (GPX)) was performed according to Duarte et al. [23]. Basically, for CAT activity, the disappearance of H_2_O_2_ was recorded at 240 nm after the addition of the vegetal extract. SOD activity was determined by oxidation of the pyrogallol at 325 nm after the addition of vegetal extract. Finally, guaiacol oxidation was measured at 470 nm after the addition of vegetal extract to determine the activity of the GPX. In the three assays, the auto-oxidation of the respective substrates was also recorded in absence of the vegetal extract.

#### Lipid peroxidation analysis

Random leaves for each plant were collected creating a pool of leaves from the same treatment using liquid nitrogen and stored until analysis at -80˚C.

To determine lipid peroxidation, MDA concentration was measured following the protocol suggested by [24]. Briefly, samples were incubated with 20% TCA containing 0.5% TBA at 95˚C for 1 h and then, samples were measured at 532 and 600 nm using a spectrophotometer to determine the MDA concentration (extinction coefficient of 156 mM^−1^ cm^−1^).

#### Oxidative stress index

The oxidative stress index (OSI) was calculated based on the results of the lipid peroxidation and the activities of the antioxidant enzymes to express the global stress in pepper plants after the experiments. This parameter was calculated following the formula described [25].

$$OSI=(\frac{\left[SOD\right]}{\left[SOD\right]0}+\frac{\left[CAT\right]}{\left[CAT\right]0}+\frac{\left[GPX\right]}{\left[GPX\right]0}+\frac{\left[MDA\right]}{\left[MDA\right]0})/4$$

In the context of this statement, [SOD], [CAT], [GPX], and [MDA] represent the respective enzyme values under different treatments applied, while [SOD]0, [CAT]0, [GPX]0, and [MDA]0 signify the control values. An index exceeding 1 suggests that the leaves experienced stress, while values below 1 suggest an absence of oxidative stress in the leaves.

## Statistical analysis

Statistical analysis was conducted using GraphPad Prism 8.4.0.671. Normality was assessed using the Kolmogorov–Smirnov test. The means of the different treatments were compared using two-way ANOVA, and statistical differences were determined using the Tukey multiple comparison test.

## Results

### Okara Enzymatic Extract Preparation

Okara is a solid organic byproduct, insoluble in water, derived from the aqueous industrial extraction of soybeans. Okara is a potential source of bioactive molecules such as peptides, isoflavones and soluble fiber but due to the insolubility it must be consider treatments that facilitate the release of its useful components. One approach to this process is the use of hydrolytic enzymes, such as proteases, from which a new soluble product, the OEE, has been obtained.

The basic chemical composition of the enzyme extract OEE is outlined in Table 1, highlighting that the primary constituent is the protein fraction at 63.4%, with carbohydrates following at 24.4%. This includes soluble fiber at 8% and insoluble fiber at 2%.

**Table 1:** Chemical composition of OEE

Total Protein	63.4 ± 1.4
Fat	2.2 ± 0.2
Carbohydrates	24.4 ± 0.9
*Soluble fiber*	8.0 ± 0.6
*Insoluble fiber*	2.0 ± 0.3
Ash	6.6 ± 0.4

(% w/w of dry matter)

**Fig. 1:**
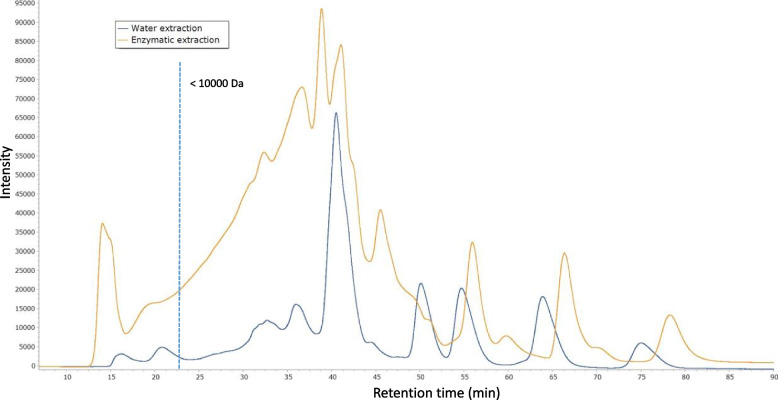
Chromatography profile of the soluble protein content of OEE according to its molecular weight using a Superdex Peptide 10/300 GL column

The protein fraction of the enzyme extract is composed of peptides < 5 kDa (Fig. 1; Table 2). Peptides are low molecular weight protein fractions with high bioactive potential.

**Table 2:** Distribution of the soluble protein content of OEE according to its molecular weight using a Superdex Peptide 10/300GL column

Molecular weight (Da)	(%)
> 1000	3.99
10,000–5000	0.83
5000–1000	3.86
< 1000	91.29

### Physiological Status in Plants

The physiological state of the plants was determined through various photosynthetic parameters such as A_N_, ETR and PhiPS2, as well as DF.

**Fig. 2:**
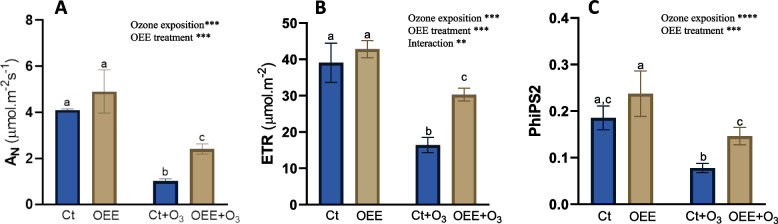
Physiological parameters. **A** A_N_; **B** ETR and **C** PhiPSII in response to O_3_ (0 and 100 ppm) under a treatment without and with OEE. Values represent mean ± SD, *n* = 5. Different letters indicate means that are significantly different from each other (two-way ANOVA, O_3_ exposition × OEE treatment; HSD test, *P* < 0.05). O_3_ exposition and OEE treatment in the corner of the panel indicate main or interaction significant effects (*P < 0.05; **P < 0.01; ***P < 0.0005; ****P < 0.0001)

After O_3_ exposure, A_N_, ETR and PhiPS2 were significantly affected (Fig. 2A-C), showing decreases of 75%, 58% and 57.8% respectively, compared to the control. Treatment with OEE didn’t significantly modify these parameters but clearly protect the decrease induced by O_3_ in all of them (51% in A_N_; 29% in ETR; 38.4% in PhiPS2).

**Fig. 3:**
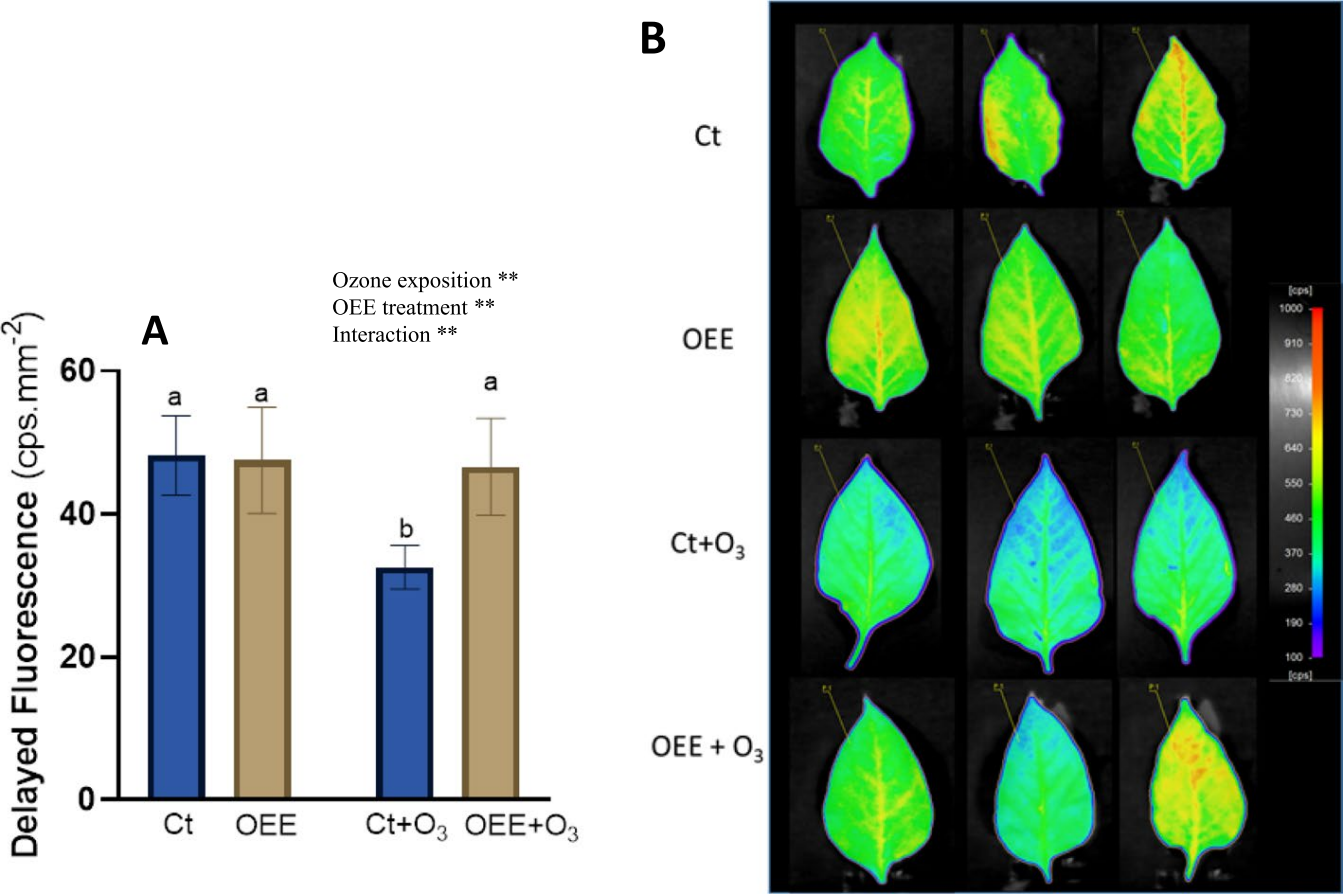
Delayed fluorescence in leaves of pepper plants in response to ozone (O3) (0 and 100 ppm) under a treatment without and with OEE). **A** Counts per second (cps) values for each treatment. Values represent mean ± SD, *n* = 5. Different letters indicate means that are significantly different from each other (two-way ANOVA, O_3_ exposition × OEE treatment; HSD test, *P* < 0.05). O_3_ exposition and OEE treatment in the corner of the panel indicate main or interaction significant effects (*P < 0.05; **P < 0.01; ***P < 0.0005; ****P < 0.0001). **B** photographs taken by the plant imaging system NightShade LB 985. Delayed fluorescence was used as a direct indicator of the chlorophyll content. The color scale reflects the detected counts per second (cps) of delayed fluorescence emission in leaves. Red colour indicates high intensities representing high chlorophyll content, blue colour indicated low intensities of fluorescence, indicating low amounts of chlorophyll

We also evaluated DF, closely link to photosynthesis reactions and thus an indicator of plant stress status. In fact DF has been used as a direct indicator of the chlorophyll content [26]. As could be visualized by the imaging and graph of Figs. 3A and B, the Ct and OEE groups showed similar DF values which indicates that OEE did not induce stress in the plants. O_3_ exposure significantly decrease DF values (26% compared to Ct; Fig. 3A) and this decreased was completely prevented by OEE (Fig. 3A).

These results suggest that OEE does not affect physiological status in plants but protect them against photosynthetic damage induced by ozone helping to maintain physiological status under this abiotic stress.

### RNAseq

To further investigate the impact of ozone on the photosynthetic machinery, the RNA expression levels of various components of photosystem II were analyzed.

**Fig. 4:**
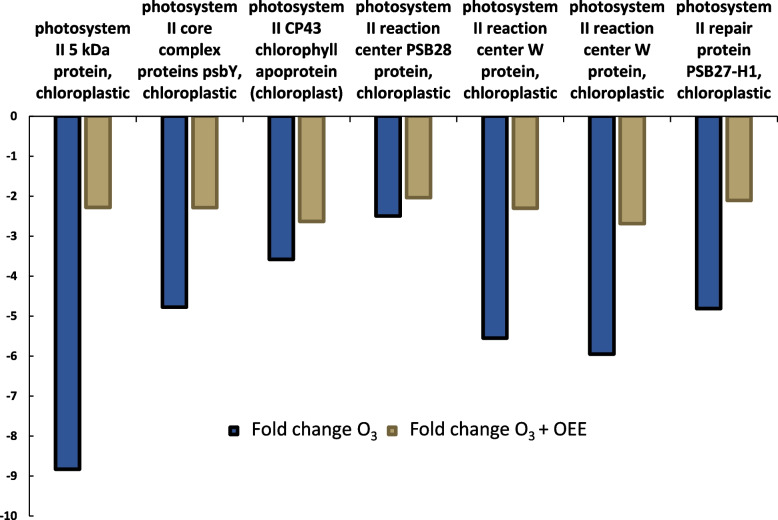
Fold-change of differentially expressed genes related to Photosystem II. Genes differentially expressed when ozone is applied to the plant are shown in blue, and in yellow when ozone plus the treatment with OEE was applied

As seen in Fig. 4, in the presence of ozone, genes related to photosystem II are repressed compared to the control group. However, plants exposed to ozone and treated with OEE show lower levels of inhibition of these genes. For example, the 5 KDa protein of photosystem II is three times less inhibited, and the W protein of the reaction center is twice as inhibited compared to plants only treated with ozone. Therefore, the OEE treatment appears to offer protection against ozone-induced damage in photosynthetic system.

### Oxidative Stress Level in Plants

**Fig. 5:**
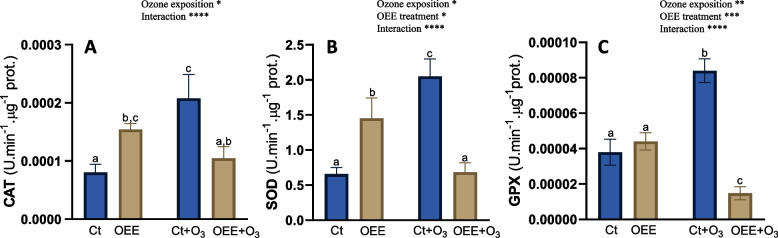
Antioxidant enzyme activities. **A** CAT **B** SOD and **C** GPX in response to O_3_ (0 and 100 ppm) under a treatment without and with OEE. Values represent mean ± SD, *n* = 5. Different letters indicate means that are significantly different from each other (two-way ANOVA, O_3_ exposition × OEE treatment; HSD test, *P* < 0.05). O_3_ exposition and OEE treatment in the corner of the panel indicate main or interaction significant effects (*P < 0.05; **P < 0.01; ***P < 0.0005; ****P < 0.0001)

To evaluate oxidative stress induced by O_3_, antioxidant enzyme activities, CAT, SOD and GPX were measured. As expected, ozone significantly induced the enzymatic activities (Fig. 5A-C), being SOD activity specially affected (increase was more than threefold). Interestingly OEE treatment also induced CAT and SOD activities in absence of ozone (87% and 121% respectively), but completely prevented the increased induced by ozone. Finally, OEE did not induce significantly the GPX activity compared with control group (Fig. 5C) although after OEE treatment, the increased induced by ozone (122%, Ct + O_3_ compared to Ct) was completely reverted.

**Fig. 6:**
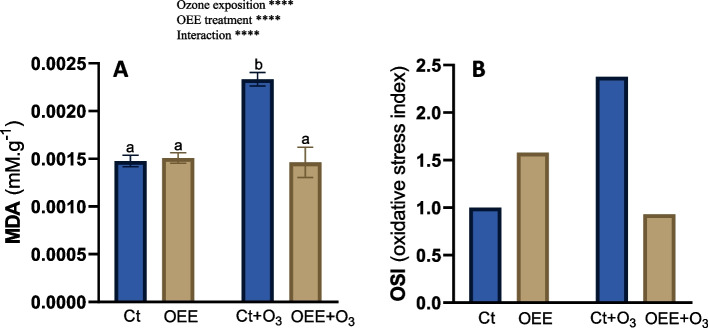
MDA content (**A**) and OSI (**B**) in leaves of pepper plants in response to ozone (O_3_) (0 and 100 ppm) under a treatment without and with OEE. Values represent mean ± SD, *n* = 5. Different letters indicate means that are significantly different from each other (two-way ANOVA, O_3_ exposition × OEE treatment; HSD test, *P* < 0.05). O_3_ exposition and OEE treatment in the corner of the panel indicate main or interaction significant effects (*P < 0.05; **P < 0.01; ***P < 0.0005; ****P < 0.0001)

MDA was measured as an indicator of lipid peroxidation due to oxidative stress [27]. As showing Fig. 6A, OEE treatment avoid the increase in MDA induced by ozone with no effect in MDA values in absence of ozone, which again underline that OEE did not induce stress in the plants.

The protective role of OEE against oxidative damage was also reflected in the OSI values (Fig. 6B). OSI values are in accordance with activities values, that show an increase after OEE treatment (1.58) but clearly protection after ozone exposition compared with control group (0.9 vs 2.37, respectively).

## Discussion

The present work demonstrates the protective effect of a biostimulant (OEE) from okara against O_3_-induced damage in peppers plants. We selected pepper plants because pepper is a vegetable crop of significant agricultural and economic importance, ranking as the second most traded spice globally. Substantial losses in pepper production often result from abiotic stresses, including ozone exposure. In fact, capsicum pepper cultivation is predominantly situated in regions where ozone concentrations are escalating to phytotoxic levels [28]. OEE protects against ozone-induced changes in photosynthetic parameters including A_N_, ETR and PhiPS2 as well as in DF while also protecting against lipid peroxidation. Given that the relief of abiotic stress is often highlighted as a prominent benefit of biostimulants [29] we propose that OEE possesses biostimulant capacity.

The chemical composition of okara is described in [18], highlighting the high level of protein and fibers. Okara is a potential source of bioactive molecules such as peptides, isoflavones and soluble fiber that are currently of great interest due to their countless benefits for both humans and agriculture [30, 31]. However the high content of insoluble biomolecules in okara can limit its direct effectiveness as a biostimulant. To employ okara in a way that improves its usefulness as a bio-stimulant, it is important to consider treatments that increase its solubility or facilitate the release of its useful components, more soluble and accessible to plants and microorganisms. As show in Fig. 1, protein hydrolysates contain peptides and amino acids resulting from enzymatic hydrolysis. They currently have great interest in agronomy due to their positive influence on growth, improvement in N absorption and assimilation, and their direct involvement in numerous metabolic processes and defense against oxidative stress by plants [32].

The adverse impacts of O_3_ on plants are extensively documented and include, among other effects, a decline in photosynthesis and the manifestation of chlorotic and necrotic spots on leaves [2, 8]. Accordingly, the present data show that the acute O_3_-exposure experimental design by us induced changes in physiological status of pepper plants, as evidenced by the decrease in parameters such as A_N_, ET and PhiPS2 (Fig. 2A-C), which is in agreement with previous report by our group [15]. Interestingly, foliar application of OEE partly reverted the O_3_-induced decreased in those parameters.

The damage inflicted on plants by O_3_ is primarily attributed to the elevated production of ROS upon its entry into the apoplastic space [33]. ROS generated by exposure to O_3_ are responsible for direct oxidative damage to various molecules involved in the photosynthetic process, such as chlorophylls a and b, and Rubisco, whose activity and content have been shown to decrease under such stress conditions [34]. Accordingly, our results show that DF was also affected (Fig. 3). Delayed fluorescence has been used as a direct indicator of the chlorophyll content. Low signals of delayed fluorescence has been described previously after biotic ozone-induced stress [15] and also in wheat leaves after biotic stress, such as infection with *S. graminum*, which suggested the occurrence of chlorophyll degradation [26]. Additionally, the harmful effects of O_3_ on photosynthetic electron transport, particularly on the function of photosystem II, have been demonstrated [35, 36]. In this context, our RNAseq results revealed that ozone represses genes related to photosystem II (Fig. 4). Interestingly OEE partly reverted ozone-induced effect on DF and gene expression.

In the cell, ROS interacts directly or indirectly with biomolecules, damaging them. Thus, ROS induces lipid peroxidation of cell membranes, denaturation of proteins, oxidation of carbohydrates, or fragmentation of pigments [37]. To counteract ROS, which are produced not only under stress but also during the normal metabolism of plant cells in processes such as mitochondrial respiration, photosynthesis and the activity of flavin-oxidoreductases, cells possess enzymatic and non-enzymatic systems that protect them from ROS. Indeed, the increase in ROS production is associated with certain antioxidant enzymes [38, 39], which are stimulated by the upregulation of antioxidant genes [38].

### Principio del formulario

To analyse the protective role of OEE against O_3_-induced damage, we examined antioxidant enzymes activities. The results presented here demonstrate that the antioxidant enzyme activities assayed (CAT, GPX, and SOD) were upregulated after ozone exposition, and this induction was significantly reversed by foliar treatment with OEE (Fig. 5). Surprisingly, OEE also stimulated enzymatic activities even in the absence of ozone. It's crucial to highlight that the production of ROS is a prevalent plant response to various stresses, encompassing both biotic and abiotic factors (as reviewed by Sewelan et al. [40]). ROS can serve as a convergence point for different signalling pathways. Within this context, it's plausible to speculate that OEE induced a mild response activating signalling pathways that contribute to coping with subsequent stress. This could be considered a hormetic-like effect. In fact, in previous work, we have already speculated that enzymatic extracts of plant origin may exert a hormetic effect by inducing antioxidant enzyme activity [15]. Interestingly, both ROS and reactive nitrogen species are frequently linked to dose–response hormesis in both plants and animals [41–43].

Hormesis is defined as “an adaptive response of biphasic dose where it responds to a stress determining factor, in which sub-doses induce stimulation and high doses induce inhibition” [44]. From a physiological standpoint, hormesis is an adaptive response activated in an organism when subjected to low levels of a stressor, accompanied by overcompensation, when the homeostasis readjustment has been interrupted [45–47]. In plants, hormesis has been elucidated through exposure to low levels of biotic or abiotic stressors, including temperature fluctuations or radiation. This exposure predisposes plants to respond to challenging conditions by activating cellular defense mechanisms [47, 48].

The mechanism of hormesis in plants is not well-defined, although it has been proposed that the induction of ROS by weak stressors may play a central role through activation of antioxidant defense systems, stress-signaling hormones, or adaptive growth responses [49]. Accordingly, it has been described that the induction of low and sub-toxic concentrations of ROS by mild stressors, such as may occurs after foliar application of OEE, has the capacity to generate a hormetic response, activating antioxidative defense and adaptive responses [49].

Biostimulants are defined as substances that promote plant growth, increase the ability to tolerate biotic or abiotic stress, and improve crop quality [50, 51]. Biostimulants are a broad group of compounds of diverse nature, including plant growth-promoting bacteria, beneficial fungi, humic acids, seaweeds, protein hydrolysates or amino acids [50–55]. In the context of induced hormesis, biostimulant activate secondary metabolism and induce genes expression to recover homeostasis [56] enabling plants to tolerate stresses [57]. So, when biostimulants are applied at right time can improved plant growth, and the simultaneous use of several biostimulants can effectively alleviate environmental impacts [58].

The enzymatic hydrolysis of okara yielding an extract rich in isoflavones [18]. Isoflavones engage in hydrophobic interactions with the proteins in which they are embedded, and treatment with proteases at alkaline pH solubilizes them. In this regard, a direct correlation has been identified between the solubilization of proteins and isoflavones in okara [18, 59], indicating that protease treatment is effective for the recovery of isoflavones. Interestingly OEE contains most of the isoflavones found in okara, with a predominant presence of beta-glucosides such as genistin, which represents about 50% of the total isoflavone content [18]. Isoflavones are the most prominent functional component of soy. They exert antioxidant activity, protect plants from diseases (such as antimicrobial and antiherbivore activities) and have positive effects on the life quality of plants [60]. In soybean, it has been described that isoflavones act as phytoalexins [61]. Phytoalexins are plant metabolites that protect plants due to potent antibacterial, antiviral effects, antiherbivore effects, and even effects in abiotic stress situations such as ozone [62–65]. We propose that isoflavones present in OEE may act as elicitors, being metabolite-inducing factors that mimic stress conditions and contribute to the hormetic-like effect in pepper plants, allowing them to cope with further abiotic stress induced by ozone.

The hormetic-like effect was also evinced in the oxidative stress index. OSI values (Fig. 6B) indicated an increase in oxidative stress after OEE treatment (1.58). However, there was a clear protection after ozone exposition when compared with control group (0.9 vs 2.37, respectively). This supports the hypothesis that OEE´s bioactive compounds induce a mild stress condition in pepper plants, which, in turn, protects them against further abiotic stress.

MDA resulting from the peroxidation of polyunsaturated fatty acids within cells is regarded as a dependable indicator for evaluating the degree of injury in stressed plants [66]. The higher the extent of damage to the plant, the greater the MDA content, as evidenced by studies on plant responses to both abiotic and biotic stresses [67]. In fact, when pepper plants are exposed to O_3_, it has been observed that biomolecules undergo damage from oxidation caused by reactive oxygen species (ROS) as well as direct interaction with O_3_. This results in a decrease in chlorophyll content and an increase in lipid peroxidation [68]. Consistent with the antioxidant activity of OEE [18] we observed that the foliar application of OEE prevented the lipid peroxidation induced by ozone, as evidenced by the MDA values (Fig. 6A). Therefore, we can assume that OEE protects plants from ozone-induced abiotic damage both through its hormetic effect and, as expected, due to its antioxidant capacity.

OEE contains different bioactives compounds that contribute to its antioxidant capacity including isoflavones. Genistein and genistin, both prevalent in OEE, have been characterized as having the most substantial antioxidant activities among all soy isoflavones [69]. Regarding the antioxidant capacity, it is essential to highlight that the protein fraction in OEE primarily consists of peptides < 1 kDa, which could contribute to the extract's antioxidant activity [18]. Treatment with proteases release releases peptides from proteins, thereby converting them into their active form [70]. These protein hydrolysates (PHs) exert multiple bioactivities, with antioxidant activity being one of the first to be recognized among their bioactive properties (for review see [71]. The bioactivities of PHs have led to considering them as a novel approach to stimulate plants, as foliar application of protein hydrolysates to plants has the potential to alleviate the impact of abiotic stressors by enhancing antioxidant capability [72]. Several studies have shown that soybean hydrolysates have the capacity to effectively counteract free radicals [73, 74]. Even more, it has been also reported antioxidant activity in soy protein derived peptides obtaining after treatment with different proteases including subtilisin from *Bacillus subtilils* [75]. The antioxidative properties of a peptide are influenced by its structure and amino acid sequence. In this regard, we have previously highlighted that the amino acid composition of OEE is characterized by a predominance of hydrophobic amino acids [18] which have been associated with the antioxidant activity of bioactive peptides [31, 74, 76, 77]. Besides, the hydrolysis of proteins results in an elevation of the concentration of sulfur amino acids, namely methionine and cysteine, both of which possess significant antioxidant potential [78]. Furthermore, the peptides in OEE have a direct relationship with isoflavones. These isoflavones are converted into their bioactive form (aglycones) after hydrolysis with proteases, with a notable concentration of genistin, a highly antioxidant aglycone [18]

Moreover, it should be also taking into account that soybean seeds and its derived okara are good sources of dietary fibers [16]. In OEE, the carbohydrates fraction represents 24.4% of the dry matter including insoluble (2%) and soluble fibers (8%) (Table 1). Okara is enriched in cell wall polysaccharides, and it has been described that certain fractions of okara polysaccharides exert antioxidant activity, with pectins or solubilized simple saccharides playing a significant role [79]. Therefore, a characterization of OEE carbohydrate fraction would be interesting.

## Conclusions

In this study, we have analyzed the protective role of an enzymatic extract obtained from okara against abiotic stress induced by O_3_. The chosen model is of interest as O_3_ is responsible for significant losses in agriculture, and in the coming years, the increase in pollutants derived from human activity, coupled with global warming, is expected to worsen these losses. Additionally, for the study, pepper plants (*Capsicum annum*) were selected since they are predominantly grown in subtropical regions globally, with major producers situated in developing countries where O_3_ concentrations are anticipated to rise more significantly than in other nations.

Consistent with its antioxidant activity, in line with the composition of bioactive compounds such as isoflavones and PHs, OEE treatment protects against ozone-induced damage, as evidenced by physiological parameters such as A_N_, ETR, PhiPS2, and DF, expression of genes related to photosystem II as well as levels of MDA. Interestingly, OEE induced a moderate oxidative stress that protected against subsequent ozone-induced damage, which can be interpreted as a hormetic effect.

Altogether, considering that promoting plant resistance against abiotic damage is a central feature of biostimulants, we propose that OEE possesses properties that make its use as a biostimulant feasible. Nevertheless, additional research is necessary to elucidate the mechanisms underlying hormetic and protective effects. Given our hypothesis that bioactive compounds in OEE, especially isoflavones, may function as elicitors, it would be valuable to explore the key pathways associated with the synthesis of secondary metabolites and defense mechanisms.

## Data Availability

All data generated or analysed during this study are included in this article.

## Abbreviations

O_3_: Ozone
ROS: Reactive oxygen species
OEE: Okara Enzymatic Extract
A_N_: Net photosynthetic rate
ETR: Electron transport rate
PhiPS2: Effective quantum yield of PSII
DF: Delayed fluorescence
MDA: Malondialdehyde
OSI: Oxidative stress index
EDU: Ethylene diurea– (N-[2-(2-oxo-1-imidazolidinyl) ethyl]-N0 phenyl urea
CAT: Catalase
SOD: Superoxide dismutase
GPX: Guaiacol peroxidase
PHs: Protein hydrolysates
